# The Transcription Factor CREB1 Triggers the Progression of Clear Cell Renal Cell Carcinoma by Promoting CENPE Expression

**DOI:** 10.1111/jcmm.70773

**Published:** 2025-08-12

**Authors:** Hao Jiang, Jingyuan Tang, Zhijun Cao, Feng Qiu, Zhuodong Chai, Jiaqian Qi, Feng Zhou, Yuhua Huang

**Affiliations:** ^1^ Department of Urology The First Affiliated Hospital of Soochow University Suzhou People's Republic of China; ^2^ Department of Urology Jiangsu Province Hospital of Chinese Medicine, Affiliated Hospital of Nanjing University of Chinese Medicine Nanjing People's Republic of China; ^3^ Department of Urology Suzhou Ninth People's Hospital, Soochow University Suzhou People's Republic of China; ^4^ Department of Pharmaceutical Sciences Irma Lerma Rangel School of Pharmacy, Texas A&M University Kingsville Texas USA

**Keywords:** ccRCC, CENPE, CREB1, EMT, progression

## Abstract

Centromere‐associated protein E (CENPE) has been identified as overexpressed in multiple cancers and exerts a tumour promotion function by affecting chromosome misalignment and mitosis. However, the expression pattern, biological roles, and underlying molecular mechanism of CENPE in clear cell renal cell carcinoma (ccRCC) progression have not been fully elucidated. In the present study, the expression levels of CENPE in ccRCC and paracancerous specimens were measured using the public RNA sequencing data and validated in a cohort of ccRCC samples from our centre. We found that CENPE was significantly over‐expressed in ccRCC tissues and promoted proliferative and metastatic abilities of ccRCC cells and xenografts through regulating the epithelial‐mesenchymal transition (EMT) process. Furthermore, bioinformatic analysis and ChIP assay indicated that the transcription factor CREB1 bound to the promoter region of CENPE and activated its transcription in ccRCC cells. Taken together, our findings demonstrated that the CREB1‐CENPE axis was responsible for stimulating the in vitro and in vivo progression of ccRCC, serving as a promising therapeutic target for ccRCC.

## 1 Introduction

Renal cell carcinoma (RCC) is a malignancy originated from the renal tubular epithelium. The high morbidity and mortality of RCC greatly threaten human lives. Globally, more than 400,000 new RCC cases are diagnosed each year, leading to over 170,000 deaths from kidney cancer annually. In 2024, it is estimated that 81,610 new cases of RCC will be diagnosed in the United States [1]. More seriously, the incidence of RCC increases by 2%–3% every decade [2]. Among the various subtypes, clear cell renal cell carcinoma (ccRCC) is the most common subtype of RCC, which is typically localised at the early stage and removed by surgical procedures [3]. Nevertheless, high recurrent and metastatic rates present a substantial challenge to improving patient outcomes. The 5‐year survival rate for metastatic ccRCC is under 20% [4]. A thorough illustration of the mechanisms underlying the pathogenesis of ccRCC and its metastases is therefore of great significance to improve the prognosis.

Centrosome‐associated protein E (CENPE) is functional to head up cellular cargo transportation using energy derived from ATP hydrolysis [5]. Latest evidence has supported that aberrantly expressed CENPE is a vital regulator involved in tumourigenesis. For instance, CENPE is overexpressed in non‐small cell lung cancer and associated with poor prognosis. CENPE regulates PD‐L1 by targeting its 3′ untranslated region (UTR), thereby affecting the immune microenvironment [6]. Moreover, Lysine‐Specific Demethylase 1 (LSD1)–mediated epigenetic reprogramming activates the transcription of CENPE, which in turn drives the progression of castration‐resistant prostate cancer [7]. Additionally, the expression level of CENPE is notably elevated in the basal subtype of breast cancer relative to other subtypes, and knockdown of CENPE impairs cell viability [8].

In case of renal cell carcinomas, Wu et al. reported that CENPE is a potential biomarker to predict the prognosis of RCC [9]. Furthermore, CENPE is found to promote the malignant behaviours of ccRCC in vitro by mediating the Wnt/β‐catenin pathway [10]. However, the molecular mechanism of CENPE in the progression of ccRCC has been revealed as just the tip of the iceberg, and the upstream regulators of CENPE remain largely unclear. Here, we explored the role of CENPE in ccRCC in vivo and in vitro, and preliminarily unveiled the upstream transcription factor that activated the expression of CENPE in ccRCC cells. Using a series of bioinformatic analyses, we first examined the expression level of CENPE in ccRCC specimens and its relationship with clinical features. We then predicted and verified the potential transcription factors (TFs) responsible for regulating CENPE expression and identified the specific binding sites involved. Finally, an in vivo mouse model was constructed to further explore the role of CENPE in the tumour growth of ccRCC. Our findings are expected to provide novel biomarkers for clinical management of ccRCC.

## 2 Materials and Methods

### 2.1 Bioinformatic Analyses

Data on CENPE expression levels in ccRCC and normal specimens were obtained from The Cancer Genome Atlas Kidney Renal Clear Cell Carcinoma (TCGA‐KIRC) dataset (https://cancergenome.nih.gov/), and GSE40435, GSE66272, and GSE46699 datasets (https://www.ncbi.nlm.nih.gov/geo/) from the Gene Expression Omnibus (GEO) database. TF binding profile was predicted using the JASPAR database.

### 2.2 Clinical Specimens of ccRCC

ccRCC specimens (*n* = 60) and corresponding paracancerous specimens (*n* = 60) were collected from patients admitted to the Urology Department, the First Affiliated Hospital of Soochow University between February 2014 and June 2018. Patients were followed up by telephone until November 2020. TNM staging of ccRCC was determined based on the Fuhrman histologic grading system. The collection and use of clinical specimens were conducted with informed consent from all patients, and the study was approved for experimental purposes by the Ethics Committee of the First Affiliated Hospital of Soochow University.

### 2.3 Inclusion Criteria

- primary, sporadic clear cell histology confirmed by two independent pathologists;
- surgical treatment with radical or partial nephrectomy between February 2014 and June 2018;
- no neoadjuvant or adjuvant systemic therapy before tissue collection;
- availability of paired tumour and adjacent non‐tumour renal cortex (> 2 cm from the margin);
- complete clinicopathological and follow‐up information.

### 2.4 Exclusion Criteria

Non‐ccRCC histology, metastatic or recurrent disease at diagnosis, prior systemic therapy, or concurrent malignancies.

### 2.5 Cell Culture and Transfection

Except for the RCC cell line CAKI‐1 cultured in McCoy's 5A medium, the remaining RCC cell lines (A498, 786O, 769P, ACHN and OSRC‐2) and normal kidney cell lines (HK‐2 and 293 T) were cultured in RPMI‐1640 (Gibco, MA, USA) containing 10% foetal bovine serum (FBS, Gibco) and penicillin–streptomycin (1: 100, Invitrogen, CA, USA) in an incubator with 5% CO_2_ at 37°C.

Stable transfection of cells with lentiviral vectors (GeneChem, Shanghai, China) was selected by 2‐week induction of puromycin 4 μg/mL.

### 2.6 qRT‐PCR

Total cellular RNA obtained by TRIzol (Invitrogen) was reversely transcribed to prepare cDNA using the PrimeScript RT reagent Kit (Takara, Shiga, Japan). The quantitative PCR was conducted using Takara Bio's SYBR Premix Ex Taq under the recommended thermal cycling conditions. Relative level of CENPE (forward: GGAGAAAGATGACCTACAGAGGC; reverse: AGTTCCTCTTCAGTTTCCAGGTG) was valued using the 2^−ΔΔCt^ method. GAPDH (forward: GTCTCCTCTGACTTCAACAGCG; reverse: ACCACCCTGTTGCTGTAGCCAA) was used as the reference gene for normalisation.

### 2.7 Western Blot

Total proteins were extracted by RIPA lysis buffer on ice. The extracted protein samples were then separated by 10% SDS‐PAGE and transferred onto PVDF membranes. Following the immersion in 5% skim milk, membranes were immunoblotted with anti‐CENPE, anti‐Vimentin, anti‐N‐cadherin, anti‐MMP2, anti‐MMP9, anti‐CREB1, anti‐β‐Catenin, anti‐GAPDH, and anti‐α‐tubulin (1:1000, Abcam, Cambridge, UK; CST, MA, USA) at 4°C overnight, and secondary antibodies (1:3000, CST) for 2 h. Enhanced chemiluminescence was used to visualise the Western blot bands, and their relative intensities (grey values) were quantified using Image J.

### 2.8 Immunohistochemical (IHC) Staining

Tissue sections (4 μm) were deparaffinised in xylene and rehydrated through a graded ethanol series. Antigen retrieval was performed by heating the sections in sodium citrate buffer (pH 6.0) in a microwave for 15 min. After cooling to room temperature, the sections were blocked with 3% hydrogen peroxide for 10 min to quench endogenous peroxidase activity. Subsequently, sections were incubated with the primary antibody against CENPE (1:100, Abcam, Cambridge, UK) or other primary antibodies overnight at 4°C. After washing with PBS, the sections were incubated with a biotinylated secondary antibody followed by streptavidin‐HRP complex for 30 min at room temperature. DAB (3,3′‐diaminobenzidine) was used as the chromogen, and haematoxylin was applied for nuclear counterstaining. Finally, sections were dehydrated, mounted, and visualised under a light microscope. Brown staining indicated positive expression of CENPE.

### 2.9 Proliferation Assays

For the CCK‐8 assay, 3 × 10^3^ cells/well were implanted in a 96‐well plate and cultured for 1–4 days. Fresh media containing CCK‐8 solution (1:10, Dojindo, Kumamoto, Japan) was added and incubated for 2 h. Optical density at 450 nm (OD_450_) was recorded by a microplate reader.

For the colony formation assay, 500 cells/well were implanted in a 6‐well plate and media was refreshed on Day 7, 11, and 14, respectively. Visible colonies containing at least 200 single cells were fixed and stained in 0.1% crystal violet for 20 min, followed by image capturing and counting.

For the EdU incorporation assay, 3 × 10^3^ cells/well were implanted in a 6‐well plate. Cells were sequentially incubated with EdU solution (50 μM) for 2 h, fixative solution for 15 min, permeabilization buffer for 15 min and reaction mix (RiboBio, Guangzhou, China) for 30 min. After the EdU labelling, Hoechst 33342 was used for nuclear staining. EdU‐positive cells (red) and Hoechst‐stained nuclei (blue) were visualised and counted under a fluorescence microscope.

### 2.10 Transwell Assay

Transwell inserts with 8 μm pore size (24‐well format, Corning, NY, USA) were used to assess cell migration and invasion. For the migration assay, 2 × 10^3^ cells suspended in serum‐free media (200 μL) were seeded into the upper chamber of the inserts, while complete medium (500 μL) was added to the lower chamber. After 48 h of cell culture, migrated cells on the lower surface of the membrane were fixed and stained for cell counting. For the invasion assay, the same procedure was followed, but the inserts were pre‐coated with Matrigel to simulate the extracellular matrix.

### 2.11 Chromatin Immunoprecipitation (ChIP)

Protein‐DNA complexes were crosslinked by cell fixation in 1% formaldehyde. Following glycine incubation and a 5‐min rocking step, the cell pellets were lysed on ice and sonicated to shear the chromatin into fragments of approximately 1 kb in length. The fragmented chromatin was centrifuged at 12,000 *g* for 10 min, and the supernatant was resuspended in 1 mL of dilution buffer. This solution was incubated with anti‐CREB1 antibody and normal IgG as a control for 15 min in a water bath. Then, magnetic beads were added to bind the antibody‐chromatin complexes. After washing to remove unbound materials, the chromatin was subjected to qRT‐PCR to analyse specific DNA sequences associated with CREB1.

### 2.12 In Vivo Xenograft Model

Animal experiments were approved by the Animal Ethics Committee of Soochow University. Subcutaneous injection of 7 × 10^6^ CAKI‐1 cells with transfection of sh‐NC (*n* = 6) and sh‐CENPE (*n* = 6) was performed in the posterior flank of 5‐week‐old female nude mice to construct the xenograft model. Tumour width (*w*) and length (*l*) were measured using a calliper every week. After 6 weeks, the mice were sacrificed to collect xenograft tumours for weighing and calculating the volume (tumour volume [mm^3^] = *w* ^2^ × *l*/2). Protein expression levels in the xenograft tumours were assessed by immunohistochemistry (IHC) as previously reported [10].

### 2.13 Dual Luciferase Reporter Activity Assay

Dual luciferase assay was performed as per standard protocols (Promega, Madison, USA). The luciferase gene was cloned downstream of the wild‐type or mutant region of the CENPE, and co‐transfected into 786O with the pcDNA3.1‐CREB1 and control vector. After 48 h, the cells were lysed and the firefly luciferase activity was measured using the Dual‐Luciferase Reporter Assay System (Promega) normalised to Renilla luciferase as the internal reference.

### 2.14 Statistical Processing

Expression levels of CENPE and its prognostic potential using online data were processed using “edgeR” package for differential expression analysis and “survival” package for survival analysis in R, respectively. Differences between groups were analysed by the independent samples *t*‐test. Kaplan–Meier curves were plotted for survival analysis, with significance determined by log‐rank tests. A *p*‐value of less than 0.05 was considered statistically significant. Continuous variables were first assessed with the Shapiro–Wilk test for normality and the Levene test for homogeneity of variance. When both assumptions were satisfied, comparisons among three or more independent groups were performed with one‐way ANOVA followed, upon significance, by Tukey's post hoc multiple‐comparison test; if either assumption was violated, the Kruskal–Wallis test was used with Dunn's post hoc comparison and Bonferroni adjustment. Two‐group comparisons employed the unpaired Student's *t*‐test (parametric) or Mann–Whitney *U*‐test (non‐parametric) as appropriate. Paired tumour‐versus‐adjacent analyses were conducted with the paired *t*‐test or Wilcoxon signed‐rank test, depending on data distribution. Survival differences were assessed by Kaplan–Meier analysis with the log‐rank test, and hazard ratios were obtained from univariate Cox regression. For high‐throughput RNA‐seq datasets, differential expression was determined with the edgeR exact test and controlled for multiple testing using the Benjamini–Hochberg procedure (false‐discovery rate < 0.05; |log_2_ fold change| > 1). All statistical tests were two‐sided, and significance was defined as *p* < 0.05 after any adjustment. Analyses were performed in R 4.3.2 (packages edgeR, survival, FSA) and GraphPad Prism 10. The specific sample sizes, biological replicates (all in vitro assays repeated independently at least three times).

## 3 Results

### 3.1 Overexpression of CENPE in ccRCC

**FIGURE 1:**
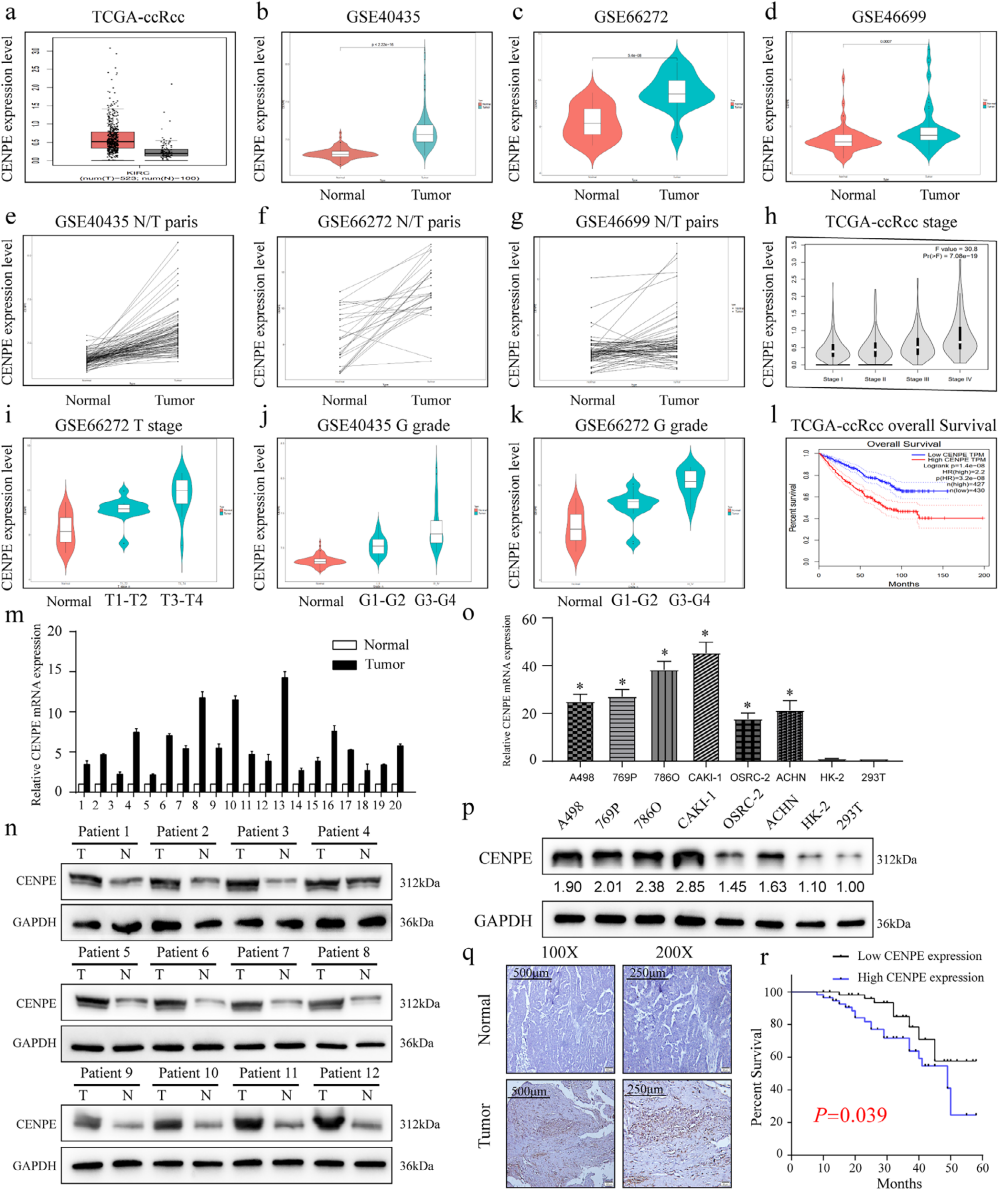
Overexpression of CENPE in ccRCC. (a) Expression levels of CENPE in the TCGA‐KIRC dataset containing 523 kidney renal clear cell carcinoma specimens and 100 normal specimens (Red: Tumour; Grey: Normal). (b–d) Expression levels of CENPE in ccRCC specimens and adjacent non‐tumoral renal specimens of the GSE40435 (b), GSE66272 (c) and GSE46699 datasets (d). (e–g) Pairwise expression levels of CENPE in ccRCC and adjacent non‐tumoral specimens of the GSE40435 (e), GSE66272 (f) and GSE46699 datasets (g). (h) Violin plots visualising CENPE levels in ccRCC specimens at stage I‐IV. (i, k) Expression levels of CENPE in ccRCC specimens classified by T staging (i) and G staging (k) using the GSE66272 dataset. (j) Expression levels of CENPE in ccRCC specimens classified by G staging using the GSE40435 dataset. (l) Overall survival of ccRCC patients stratified by the expression level of CENPE (HR, Hazard Ratio). (m, n) Expression levels of CENPE in ccRCC specimens and paired non‐tumoral specimens collected in our centre. (o, p) Expression levels of CENPE in ccRCC cell lines and normal cell lines, the numbers below represent the relative CENPE expression normalised to GAPDH, with 293 T cells set as the reference (1.00). (q) Immunohistochemical staining of CENPE in ccRCC specimens and paired non‐tumoral specimens collected in our centre (magnification = 100× and 200×), with brown staining indicates positive expression of CENPE, while blue staining (haematoxylin) marks the cell nuclei. (r) Kaplan–Meier survival of ccRCC patients classified by the median level of CENPE. *p < 0.05.

In the TCGA‐KIRC dataset, CENPE expression was found to be significantly upregulated in ccRCC specimens compared to normal controls (Figure 1a). Similarly, overexpression of CENPE was also observed in ccRCC specimens across the GSE40435, GSE66272, and GSE46699 datasets (Figure 1b–d). Paired analyses of normal and tumour tissues in these datasets further confirmed the consistent overexpression of CENPE in ccRCC tissues (Figure 1e–g). When classified by tumour staging and TNM system, high‐level CENPE was correlated with more advanced disease. Specifically, CENPE overexpression correlated with higher T stages (T3‐T4) and more severe histopathological grades (G3‐G4) in both the GSE66272 and GSE40435 datasets (Figure 1h–k). These findings suggest that increased CENPE expression is linked to more aggressive ccRCC phenotypes.

We further validated the expression level of CENPE in ccRCC specimens collected in our centre. Consistent with the public datasets, CENPE was significantly upregulated in tumour tissues compared to paracancerous tissues (Figure 1m,n). Immunohistochemical staining results (Figure 1q) revealed that CENPE expression was minimal in normal kidney tissues, while ccRCC tumour tissues exhibited strong positive staining for CENPE, indicating significant overexpression of the protein in the tumour. Additionally, the tumour tissue displayed a higher cellular density compared to the normal kidney tissue. In vitro upregulation of CENPE was also observed in multiple ccRCC cell lines compared to normal kidney cell lines (Figure 1o,p). Survival analysis indicated that higher CENPE expression was associated with poorer overall survival in ccRCC patients, as demonstrated by the TCGA‐KIRC dataset (Figure 1l). This was further validated in our cohort, where high CENPE expression correlated with significantly worse prognosis (*p* = 0.039, Figure 1r). These findings suggest that CENPE overexpression serves as an unfavourable prognostic marker in ccRCC (Figure 1l,r).

### 3.2 CENPE Promotes the Proliferation of ccRCC In Vitro

**FIGURE 2:**
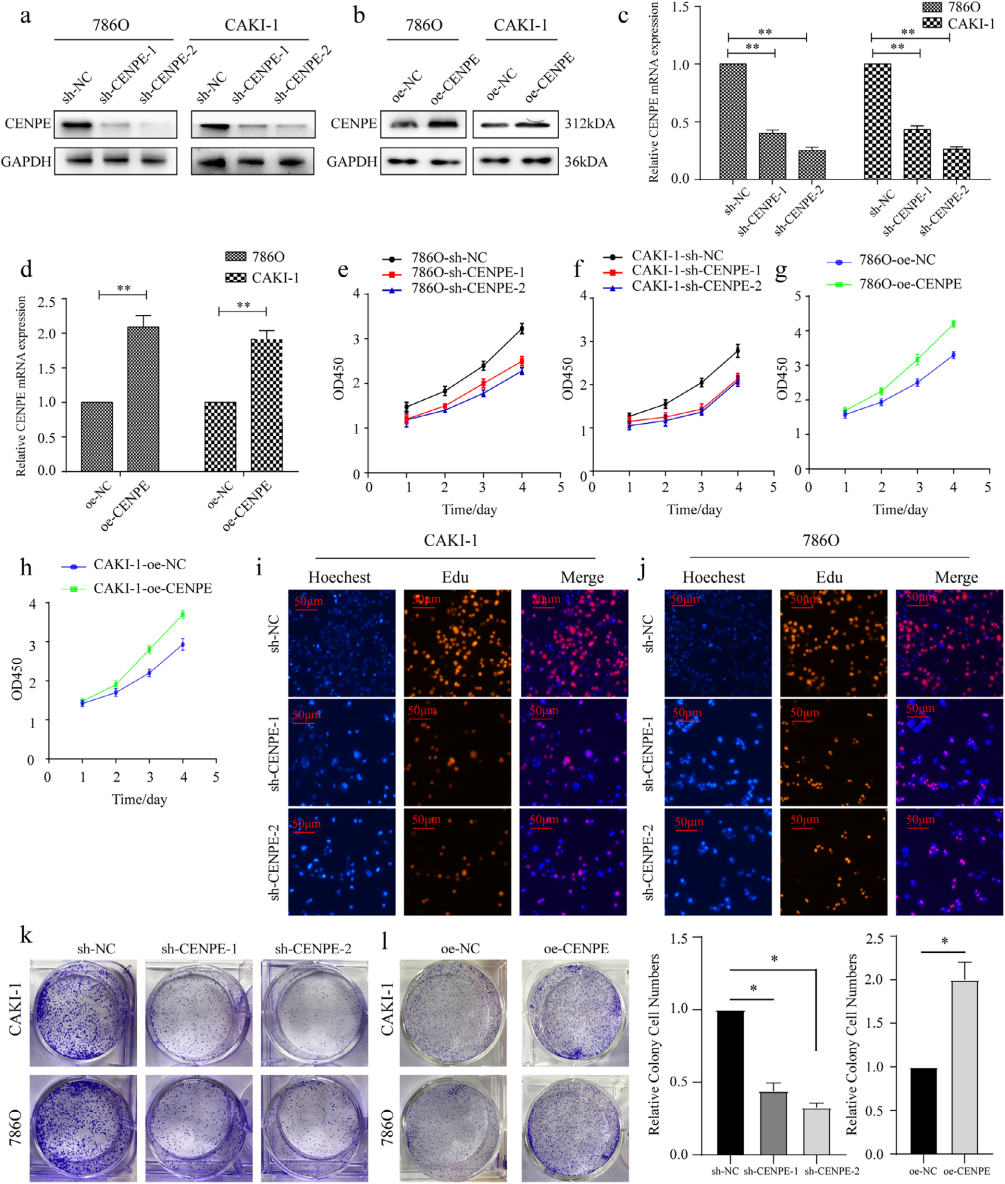
CENPE promotes the proliferation of ccRCC in vitro. (a, b) Transfection efficacy of sh‐CENPE‐1, sh‐CENPE‐2 (a) and oe‐CENPE (b) in 786O and CAKI‐1 cells detected by Western blot. (c, d) Transfection efficacy of sh‐CENPE‐1, sh‐CENPE‐2 (c) and oe‐CENPE (d) in 786O and CAKI‐1 cells detected by qRT‐PCR. (e, f) Cell viability in 786O (e) and CAKI‐1 cells (f) transfected with sh‐CENPE‐1 or sh‐CENPE‐2 detected by CCK‐8 assay. (g, h) Cell viability in 786O (g) and CAKI‐1 cells (h) transfected with oe‐CENPE detected by CCK‐8 assay. (i, j) EdU‐positive cells (red) and Hoechest‐staining nuclei (blue) in CAKI‐1 (i) and 786O cells (j) transfected with sh‐CENPE‐1 or sh‐CENPE‐2 (scale bar = 50 μm). (k, l) Colony formation in CAKI‐1 and 786O cells transfected with sh‐CENPE‐1 or sh‐CENPE‐2 (k) and oe‐CENPE (l). (m) Bar plots show the relative colony numbers in 786O cells following CENPE knockdown or overexpression. *p < 0.05, **p < 0.01.

786O and CAKI‐1 cells were used in the following experiments due to the enriched expression level of CENPE. Transfection of both sh‐CENPE‐1 and sh‐CENPE‐2 significantly knocked down the mRNA and protein levels of CENPE in 786O and CAKI‐1 cells (Figure 2a,c). Conversely, overexpression of CENPE (oe‐CENPE) significantly increased CENPE expression, verifying the transfection efficacy (Figure 2b,d). The role of CENPE in cell proliferation was then examined. Knockdown of CENPE remarkably reduced cell viability (Figure 2e,f), lowered the proportion of EdU‐positive cells (Figure 2i,j) and decreased colony formation numbers (Figure 2k) in ccRCC cells. On the contrary, overexpression of CENPE effectively enhanced cell viability (Figure 2g,h) and increased colony number (Figure 2l), indicating that CENPE promotes cell proliferation in ccRCC.

### 3.3 CENPE Promotes the Metastasis of ccRCC In Vitro

**FIGURE 3:**
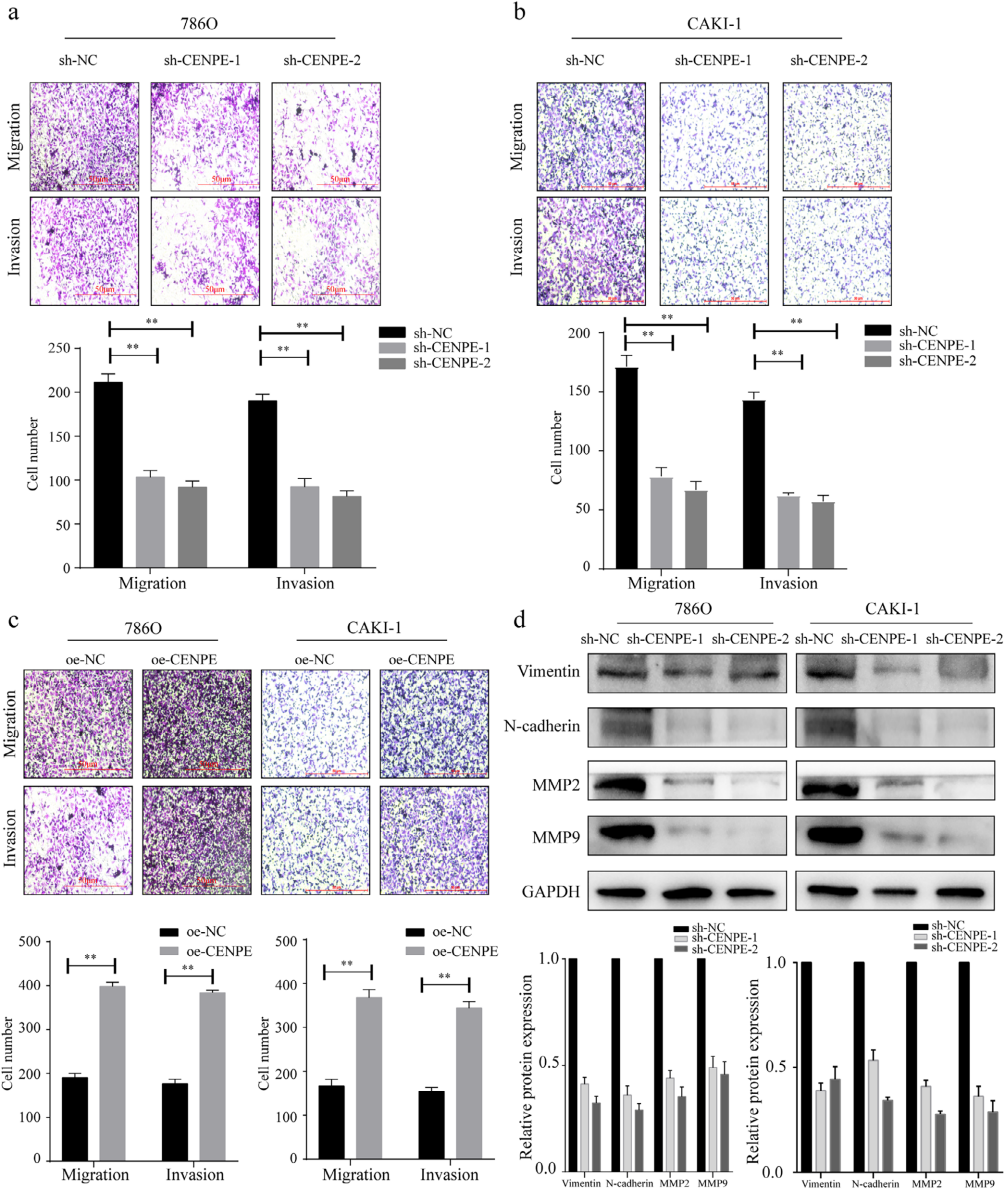
CENPE promotes the metastasis of ccRCC in vitro. (a, b) Migration and invasion assays in 786O (a) and CAKI‐1 cells (b) transfected with sh‐CENPE‐1 or sh‐CENPE‐2 (scale bar = 50 μm). (c) Migration and invasion assays in 786O and CAKI‐1 cells transfected with oe‐CENPE (scale bar = 50 μm). (d) Protein expressions of Vimentin, N‐cadherin, MMP2 and MMP9 in 786O and CAKI‐1 cells transfected with sh‐CENPE‐1 or sh‐CENPE‐2, analysed by Western blot. **p < 0.01.

CENPE knockdown significantly reduced the number of migrated and invaded cells in both 786O and CAKI‐1 cells (Figure 3a,b). Conversely, overexpression of CENPE resulted in increased migration and invasion in these ccRCC cells (Figure 3c). Additionally, the knockdown of CENPE led to the downregulation of metastasis‐related proteins Vimentin, N‐cadherin, MMP2, and MMP9. Vimentin and N‐cadherin are key markers of the epithelial–mesenchymal transition (EMT), a process that enhances cell motility and invasiveness, while MMP2 and MMP9 are matrix metalloproteinases involved in the degradation of the extracellular matrix, facilitating tumour invasion and metastasis. The decrease of these proteins indicates the inhibition of the EMT process in vitro (Figure 3d) whereas their overexpression significantly promotes the EMT process (Figure S1).

### 3.4 CREB1 Activates the Transcription of CENPE in ccRCC

**FIGURE 4:**
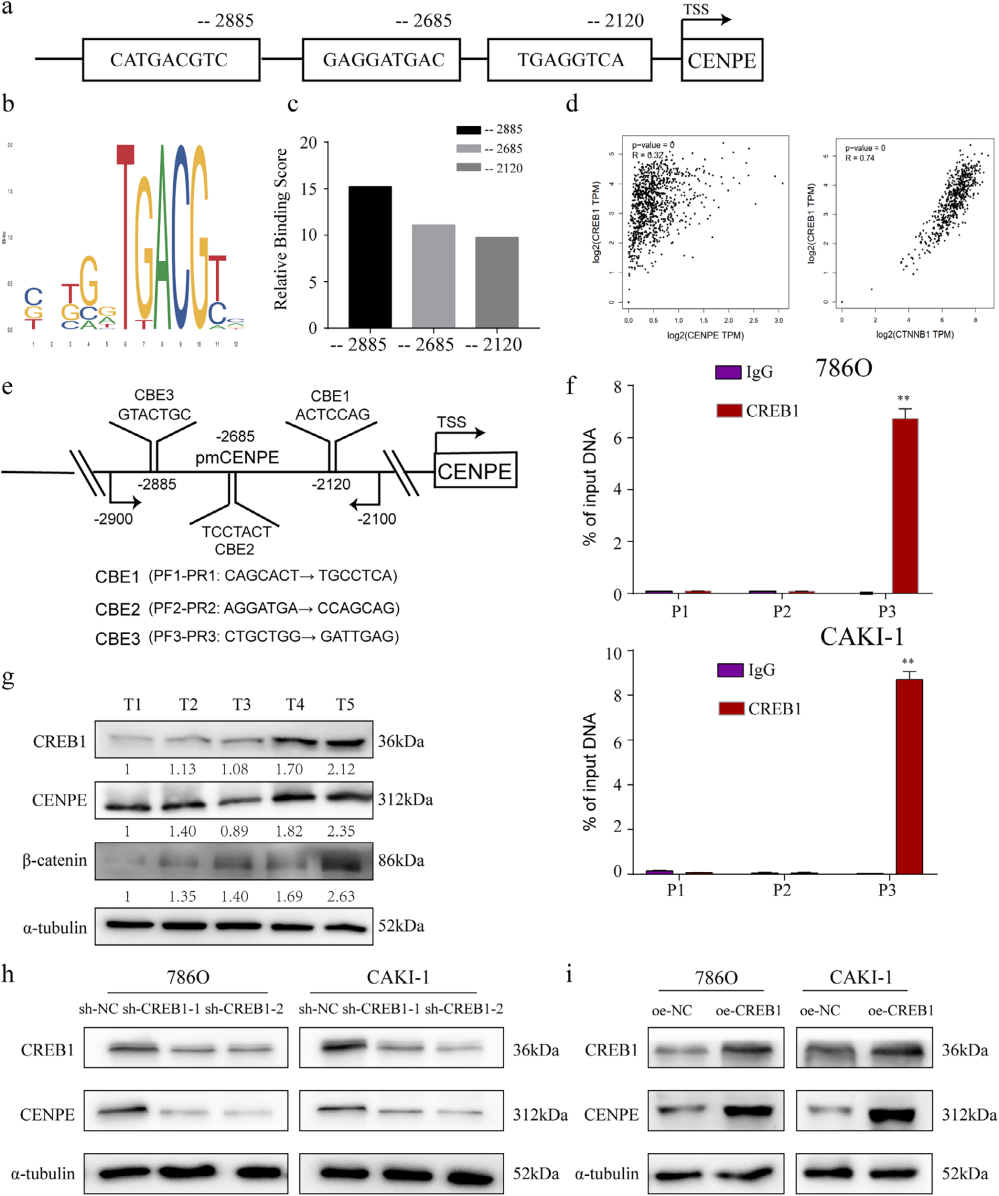
CREB1 activates the transcription of CENPE in ccRCC. (a, b) CREB1 binding sites in the promoter region of CENPE predicted by the JASPER database (a) and the binding region within the predicted binding site (b). (c) Relative score for each potential binding sequence of CENPE combined with CREB1. (d) Correlations of CREB1 with CENPE and CTNNB1 (β‐catenin) in the TCGA dataset. (e) Schematic of the CENPE promoter region showing the designed primers (CBE1, CBE2, CBE3) for ChIP assay. (f) ChIP assay visualising the input of CREB1 and IgG in the promoter region of CENPE (−2885). (g) Protein expressions of CREB1, CENPE and β‐catenin in ccRCC specimens. (h) Protein expressions of CREB1 and CENPE in 786O and CAKI‐1 cells transfected with sh‐CENPE‐1 or sh‐CENPE‐2. (i) Protein expressions of CREB1 and CENPE in 786O and CAKI‐1 cells transfected with oe‐CREB1. **p < 0.01.

Using the JASPAR database, we predicted multiple potential transcription factors that could bind to the promoter region of CENPE. Among these candidates, CREB1 was identified as a strong candidate due to its biological relevance and potential role in tumour progression. We depicted illustrations of CREB1 target sites for CENPE binding in the promoter region (Figure 4a). Further analysis of the predicted binding sequences revealed key conserved bases critical for CREB1 binding (Figure 4b), and the relative scores for each sequence of CENPE combined with CREB1 were shown in Figure 4c. To assess the relationship between CREB1 and CENPE, we performed correlation analysis using the TCGA datasets, and we discovered a positive correlation of CREB1 level with CENPE and CTNNB1 (β‐catenin) (Figure 4d), suggesting a potential regulatory relationship between these genes.

To validate CREB's role as a transcriptional regulator of CENPE, three primers, namely CBE1 (CREB1 binding ending 1), CBE2, and CBE3, were designed based on the CREB1 sequence analysed from the JASPAR database (Figure 4e). ChIP assay further demonstrated that CREB1 binds to the CENPE promoter at the −2885 site in ccRCC cells (Figure 4f). Additionally, luciferase reporter vectors containing the wildtype (WT) and mutant (MUT) CREB1 binding sequences of the CENPE were constructed. The result showed that the upregulated CREB1 remarkably increased the luciferase activity of the WT vector compared with the MUT vector (*p* < 0.001, Figure S2). Further, we also observed that CREB1 expression was found to be elevated along with increased levels of CENPE and β‐catenin from the ccRCC tissue samples in our centre (Figure 4g). Functional assays showed that CREB1 knockdown led to decreased CENPE expression, while overexpression of CREB1 significantly upregulated CENPE in ccRCC cells (Figure 4h,i). Overall, we have identified CREB1 as a positive transcriptional regulator for CENPE in ccRCC in vitro, directly binding to its promoter region and promoting its expression, thereby contributing to the upregulation of CENPE in tumour progression.

### 3.5 CREB1 Is Essential for the Oncogenic Role of CENPE in ccRCC

**FIGURE 5:**
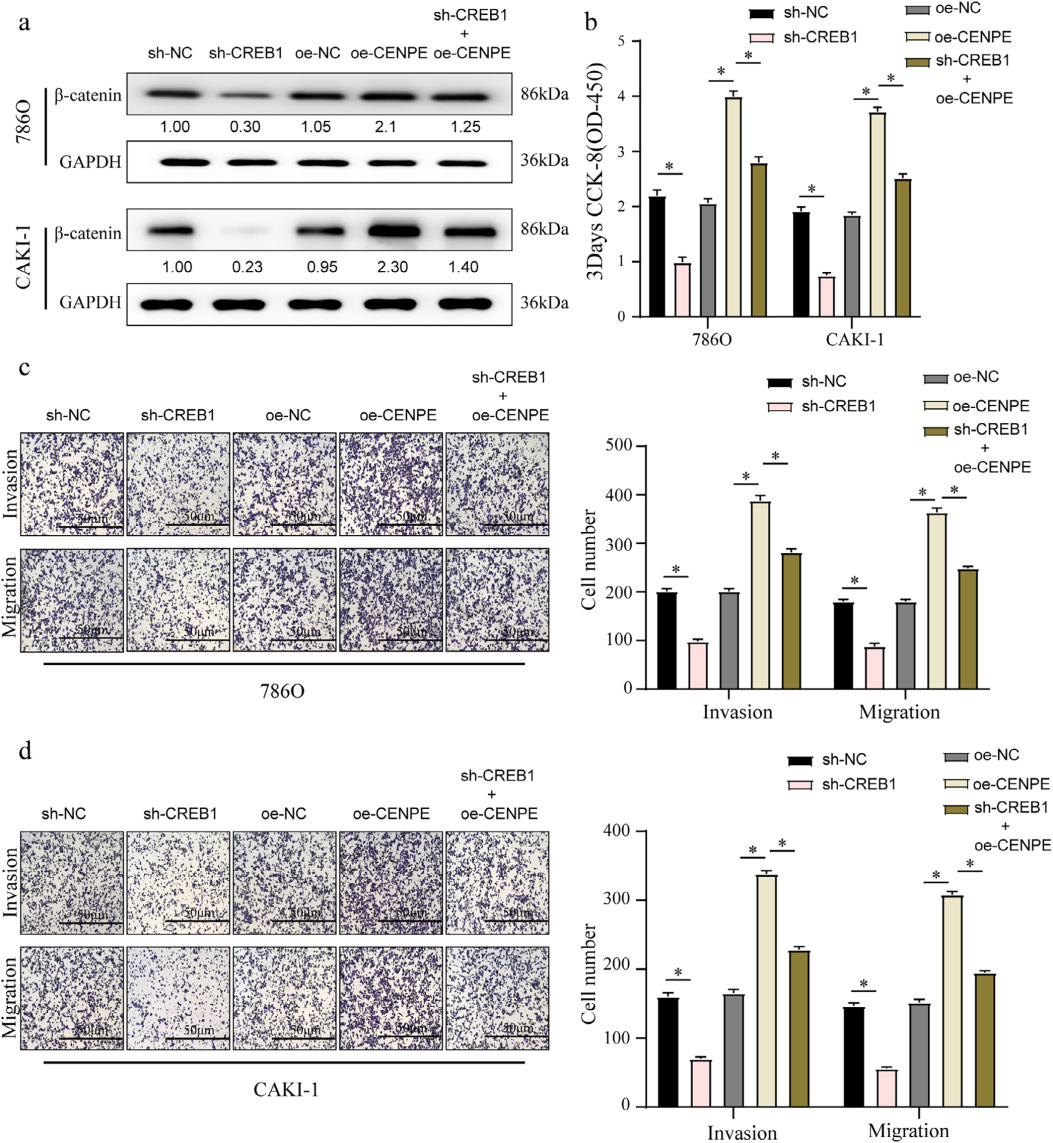
CREB1 is essential for the oncogenic role of CENPE in ccRCC. Protein expression of β‐catenin (a), cell viability (b), migration (c) and invasion (d) in 786O and CAKI‐1 cells treated with blank control, or transfected with sh‐CREB1, oe‐CENPE or sh‐CREB1 + oe‐CENPE. Scale bar = 50 μm. *p < 0.05.

In ccRCC cells, CENPE overexpression significantly upregulated β‐catenin levels, a key regulator of the Wnt/β‐catenin signalling pathway (Figure 5a). However, CREB1 knockdown markedly reduced β‐catenin expression (Figure 5a). In the co‐transfection experiments, where CENPE was overexpressed alongside CREB1 knockdown, the upregulation of β‐catenin induced by CENPE was largely neutralised by CREB1 silencing, indicating that CREB1 is a critical mediator of CENPE‐driven β‐catenin activation (Figure 5a).

In functional assay, the enhanced cell viability (Figure 5b), migrative and invasive capacities (Figure 5c,d) of ccRCC cells were observed in CENPE‐overexpressing and CREB1‐silencing 786O and CAKI‐1 cells. Notably, when CREB1 was silenced, these oncogenic effects of CENPE were significantly diminished, further supporting the essential role of CREB1 in mediating CENPE's tumour‐promoting effects in ccRCC. We therefore believed that CENPE‐induced in vitro progression of ccRCC was greatly attributed to CREB1.

### 3.6 The CREB1‐CENPE Axis Favours In Vivo Growth of ccRCC

**FIGURE 6:**
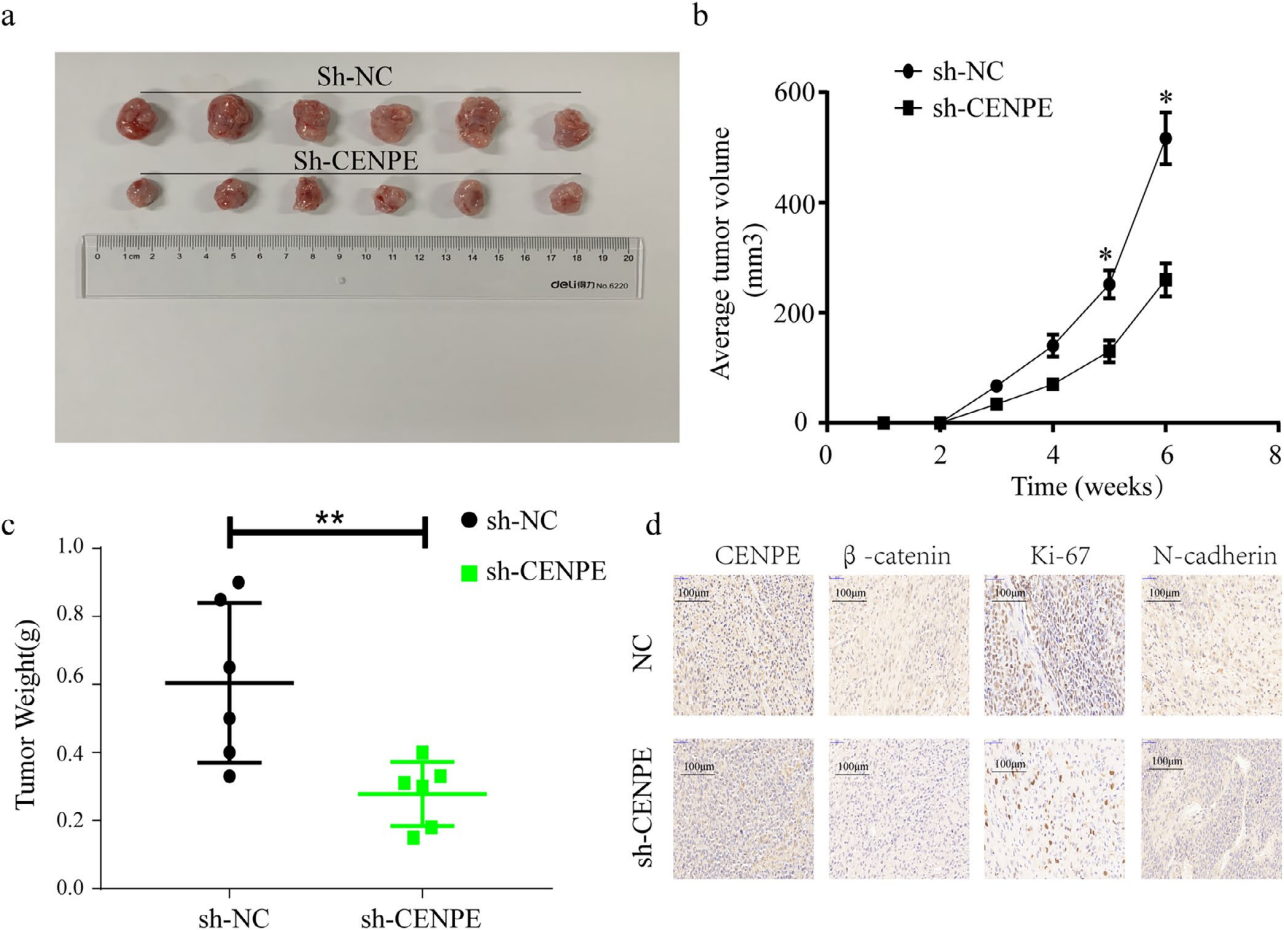
The CREB1‐CENPE axis favours in vivo growth of ccRCC. Five‐week‐old female nude mice were subcutaneously injected with 7 × 10^6^ CAKI‐1 cells transfected with sh‐NC (*n* = 6) and sh‐CENPE (*n* = 6) into the posterior flank. (a) Representative images of xenograft tumours derived from CAKI‐1 cells transfected with sh‐NC (control) or sh‐CENPE after 6 weeks of growth. (b) Average tumour volume measured at indicated time points. (c) Tumour weight of excised xenografts at the end of the experiment. (d) Immunohistochemical staining of CENPE, β‐catenin, Ki‐67 and N‐cadherin in ccRCC xenografts. *n* = 6. Scale bar = 50 μm. *p < 0.05, **p < 0.01.

To evaluate the role of the CREB1‐CENPE axis in ccRCC progression in vivo, xenograft models were established by subcutaneously injecting nude mice with CAKI‐1 cells transfected with either sh‐CENPE or control shRNA (sh‐NC). After 6 weeks of xenograft tumour growth, mice were sacrificed and the tumours were excised for analysis. A gross view of collected tumours obviously revealed a smaller size in mice injected with CAKI‐1 cells transfected with sh‐CENPE than that of the negative control (Figure 6a). Quantitative analysis revealed that both tumour volume (Figure 6b) and tumour weight (Figure 6c) were significantly reduced in the sh‐CENPE group compared to the control, demonstrating that CENPE knockdown effectively suppressed tumour growth in vivo. Furthermore, IHC staining showed reduced expressions of CENPE, β‐catenin, Ki‐67 (a proliferation marker) and N‐cadherin in mouse tumour tissues with CENPE knockdown (Figure 6d). These findings indicated that the CREB1‐CENPE axis was responsible for triggering the in vivo growth and progression of ccRCC.

## 4 Discussion

Considering the high mortality and recurrence of ccRCC, effective biomarkers to predict its progression and prognosis are urgently needed [11, 12]. In this study, we demonstrated that CENPE plays a key role in promoting the tumorigenic and metastatic properties of ccRCC. We also identified CENPE as an important oncogenic regulator in ccRCC by bioinformatics analysis of public profiling data, revealing that CENPE was found to be overexpressed in ccRCC tissues, and its elevated expression was associated with advanced tumour stages and poor clinical outcomes. Furthermore, we confirmed the over‐expression of CENPE in ccRCC in a cohort of ccRCC and normal renal tissue samples from our centre. Of importance, highly expressed CENPE is positively associated with poorer prognosis and shorter survival of ccRCC patients. As well as our findings, a large body of evidence indicated that CENPE is up‐regulated in a variety of cancers and exerts a cancer‐promoting function [13, 14, 15]. CENPE is a motor protein, and its abnormal expression has been associated with various cancers. Our study supports its role as a driver of tumorigenesis in ccRCC. CENPE has been implicated in the progression of cancers such as non‐small cell lung cancer, prostate cancer, and breast cancer [7, 8, 16, 17]; however, research on its role in ccRCC remains limited and our study is among the first to uncover the oncogenic role of CENPE in ccRCC. He et al. consistently reported an association of CENPE with adverse clinical features of hepatocellular carcinoma [18]. Meanwhile, CENPE has been found to be involved in the process of mitosis [5] and silence of CENPE greatly blocks lung adenocarcinoma proliferation [19].

Here, we also found that CENPE knockdown remarkably impaired the proliferative and invasive capacities. Invasion and metastases are fatal events for malignancies, and EMT is a prerequisite process of tumour cells to gain the mesenchymal phenotype and transitional state [20]. Moreover, the abilities of tumour cells in the cross‐talk with extracellular matrix and angiogenic factors are greatly attributed to the process of EMT [21]. Furthermore, our functional analysis demonstrated that silencing CENPE significantly impaired the viability, migration, and invasion of ccRCC cells, indicating that CENPE is a critical oncogenic factor in ccRCC. Upon CENPE knockdown, EMT‐related proteins such as Vimentin and N‐cadherin, as well as matrix metalloproteinases MMP2 and MMP9, were downregulated, further supporting CENPE's role in driving the metastatic phenotype of ccRCC. These results are consistent with previous reports suggesting that CENPE is involved in regulating cell cycle progression and migration in other types of cancer [9, 18, 22].

Accumulating evidence has supported the critical roles of TFs in malignant behaviours of RCC [23]. Zhang et al. illustrated a TF in triggering the aggravation of ccRCC as an oncogenic driver and validated it in a mouse model [24]. Through a series of bioinformatic analyses, we have confirmed that CREB1 (−2885 promoter site) was the potential TF that could regulate CENPE transcription, and it was positively correlated with CENPE and β‐catenin in ccRCC. CREB1 is a recognised TF activated by phosphorylation at Ser133 [25]. CREB1 (cAMP response element‐binding protein) is a well‐characterised transcription factor involved in regulating gene expression in response to various cellular stimuli, including stress, growth factors and hormones [26, 27]. As a recognised cancer target, CREB1 is usually upregulated in cancer tissues [28, 29]. Wang et al. similarly demonstrated the importance of CREB1 in regulating EMT in RCC through controlling MMPs [30]. In the present study, we first demonstrated that CREB1 stimulated CENPE transcription in ccRCC cells via a direct binding. Knockdown of CREB1 not only reduced the expression of CENPE but also significantly diminished the oncogenic effects of CENPE, such as enhanced cell proliferation, migration, and invasion. This indicates that CREB1 is a critical mediator of CENPE‐driven tumorigenesis in ccRCC. Importantly, we identified for the first time that the transcription factor CREB1 is a key upstream regulator of CENPE in ccRCC. CENPE‐driven stabilisation of β‐catenin enhances transcription of Cyclin D1 and c‐Myc, thereby accelerating G1/S transition and metabolic re‐programming—two hallmarks that explain the heightened proliferation we observe in vitro and in vivo.

Here, we provide evidence that CENPE promotes the proliferation and metastasis of ccRCC cells by enhancing the Wnt/β‐catenin signalling pathway, which is well known for its role in promoting tumour growth and metastatic spread. The Wnt/β‐catenin pathway has been shown to be aberrantly activated in various cancers, including renal cell carcinoma, contributing to increased cell proliferation and invasiveness [31, 32, 33, 34]. Our findings extend this knowledge by identifying that CENPE upregulates β‐catenin levels in ccRCC cells, promoting tumour progression. Interestingly, our results also indicate that knockdown of CREB1 largely neutralised the CENPE‐induced upregulation of β‐catenin, further supporting the role of the CREB1‐CENPE axis in activating the Wnt/β‐catenin signalling pathway in ccRCC. These findings underscore the importance of CREB1 in promoting tumourigenesis by regulating key oncogenes such as CENPE. Consistent with reports in non‐small‐cell lung cancer, prostate cancer and triple‐negative breast cancer [7, 8], we show that CENPE drives proliferation and invasion. All studies—including ours—link CENPE to mitotic fidelity or EMT‐related programmes, suggesting a conserved oncogenic “tool‐kit.”

According to the previous research reports, activated became phosphorylated CREB1 that played a significant biological function in tumours. Zhang et al. proved that high p‐CREB1staining was an independent risk factor for cancer‐specific free survival, overall survival and progression‐free survival in ccRCC [35]. Kim et al. demonstrated that SETD2‐mediated trimethylation of H3K36 (H3K36me3) and CREB1 phosphorylation are critical for cellular sensitivity to cisplatin in metastatic non‐small cell lung cancer [36]. Tumour microenvironment and immune escape play an important role in tumour carcinogenesis. Kim et al. revealed that in KRAS and TP53 double‐mutant pancreatic cancer, CREB1 serves as a key mediator of the interaction between the two oncogenic mutants. Upon activation, CREB1 suppresses the STING‐IRF3 signalling pathway, leading to reduced secretion of type I interferon. This impaired the infiltration and function of CD8+ T cells within the tumour microenvironment, ultimately promoting immune escape and tumour metastasis [37]. Although our study highlights CREB1 as a critical transcriptional regulator of CENPE in ccRCC, contributing to tumour progression and metastasis, research has shown that CREB1 plays a dual role in cancer, functioning as both an oncogene and a tumour suppressor depending on the cancer type, cellular context, and regulatory networks involved. Future studies should aim to further elucidate the mechanisms underlying CREB's dual role across different cancer types, which could pave the way for more precise and effective cancer treatments tailored to the unique molecular profiles of individual tumours.

Our in vivo studies using a xenograft mouse model further support the critical role of the CREB1‐CENPE axis in ccRCC progression. CAKI‐1 cells transfected with sh‐CENPE led to significantly smaller tumours in nude mice compared to the control group, with reductions observed in both tumour volume and tumour weight. Immunohistochemical (IHC) analysis of the xenograft tissues showed decreased expression of the proliferation marker Ki‐67, along with reduced levels of N‐cadherin, further supporting the notion that CENPE promotes tumour growth by enhancing cell proliferation and EMT. We finally proved the role of the CREB1‐CENPE axis in the in vivo growth of ccRCC via mediating EMT.

Identifying the CREB1‐CENPE axis in ccRCC has significant clinical implications. Given the role of CREB1 in regulating CENPE, therapies that disrupt the CREB1‐CENPE axis may be particularly effective in inhibiting tumour growth and metastasis in ccRCC patients. Currently, treatment options for ccRCC, particularly in advanced and metastatic cases, are often limited by the development of resistance to targeted therapies and immunotherapies [38, 39]. CENPE inhibitors disrupt mitotic spindle assembly, selectively targeting rapidly dividing cancer cells. Clinical trials have explored their use in hematologic malignancies and solid tumours [40, 41]. Concurrently, CREB1 inhibitors (666‐15) have been tested preclinically that inhibited CREB1 activity by disrupting its interaction with coactivators, showing efficacy in models of osteoarthritis and viral infections [42]. The development of novel drugs targeting CENPE or the CREB1‐CENPE axis may offer new therapeutic avenues, potentially overcoming some of the limitations of existing treatments. Moreover, combining CENPE inhibitors with current therapies could enhance treatment efficacy and reduce the likelihood of resistance.

The xenograft mouse model used in this study involved the subcutaneous injection of human ccRCC cells into immunodeficient mice. While these models are widely used and allow for the examination of tumour growth and progression in a controlled environment, they lack the immune system components that play a critical role in human cancer biology. In future studies, the use of humanised mouse models or syngeneic mouse models with intact immune systems would be valuable to better understand the roles of CENPE and CREB1 in regulating immune responses within ccRCC. Orthotopic models that more closely mimic the natural tumour environment could provide deeper insights into how CENPE and CREB1 promote tumour progression in a more physiologically relevant context.

First‐generation CENPE inhibitors (e.g., GSK923295, PF‐2771) have reached phase I with tolerable myelosuppression, and nanomolar CREB1 blockers such as 666‐15—or forthcoming PROTAC degraders—make the CREB1‐CENPE axis pharmacologically tractable in ccRCC. Dual targeting could deepen responses to VEGFR TKIs, convert β‐catenin–driven “cold” tumours into ICB‐sensitive ones, and complement HIF‐2α inhibitors like belzutifan. We therefore advocate pre‐clinical testing of GSK923295 or 666‐15, alone and with axitinib or anti‐PD‐1, alongside biomarker‐guided optimisation to minimise marrow toxicity.

In conclusion, our findings suggest that CENPE is a critical driver of ccRCC progression, while CREB1 plays an essential role in regulating CENPE expression. The CREB1‐CENPE axis represents a promising target for therapeutic intervention in ccRCC, and further exploration of this pathway may lead to the development of new treatment strategies for this challenging malignancy.

## 5 Conclusion

This study identified CENPE as a key promoter of ccRCC progression, driving tumour proliferation, migration, and invasion both in vivo and in vitro. Elevated CENPE expression was associated with advanced tumour stages and lower patient survival, highlighting its potential as a prognostic marker. We also discovered that CREB1 acts as an upstream regulator of CENPE, and that the CREB1‐CENPE axis promotes ccRCC progression, in part through activation of the Wnt/β‐catenin signalling pathway. These findings suggest that targeting the CREB1‐CENPE axis and its downstream signalling could offer a promising therapeutic strategy for ccRCC, opening new avenues for potential treatment options in this malignancy.

## Author Contributions

**Hao Jiang:** data curation (equal), formal analysis (equal), software (equal), validation (equal), writing – original draft (equal). **Jingyuan Tang:** software (equal), validation (equal). **Zhijun Cao:** data curation (equal), formal analysis (equal), writing – original draft (equal). **Feng Qiu:** data curation (equal), investigation (equal), methodology (equal). **Zhuodong Chai:** software (equal), supervision (equal). **Jiaqian Qi:** validation (equal), visualization (equal). **Feng Zhou:** software (equal), supervision (equal), writing – review and editing (equal). **Yuhua Huang:** conceptualization (equal), supervision (equal), validation (equal), visualization (equal), writing – review and editing (equal).

## Conflicts of Interest

The authors declare no conflicts of interest.

## Supporting information


**Figure S1:** Protein expressions of Vimentin, N‐cadherin, MMP2 and MMP9 in 786O and CAKI‐1 cells transfected with oe‐CENPE, analysed by Western blot. *p < 0.05, **p < 0.01.

**Figure S2:** Fluorescence activity in 786O cells with CENPE wildtype and mutant promotor with CREB1 pcDNA3. (***p < 0.001).

## Data Availability

All data supporting the findings of this study are available from the corresponding author upon reasonable request.
