# Supplementary figures and images for: The Transcription Factor CREB1 Triggers the Progression of Clear Cell Renal Cell Carcinoma by Promoting CENPE Expression
Source: J Cell Mol Med. 2025 Aug 12;29(15):e70773. doi: 10.1111/jcmm.70773 (PMC12341428; doi:10.1111/jcmm.70773)

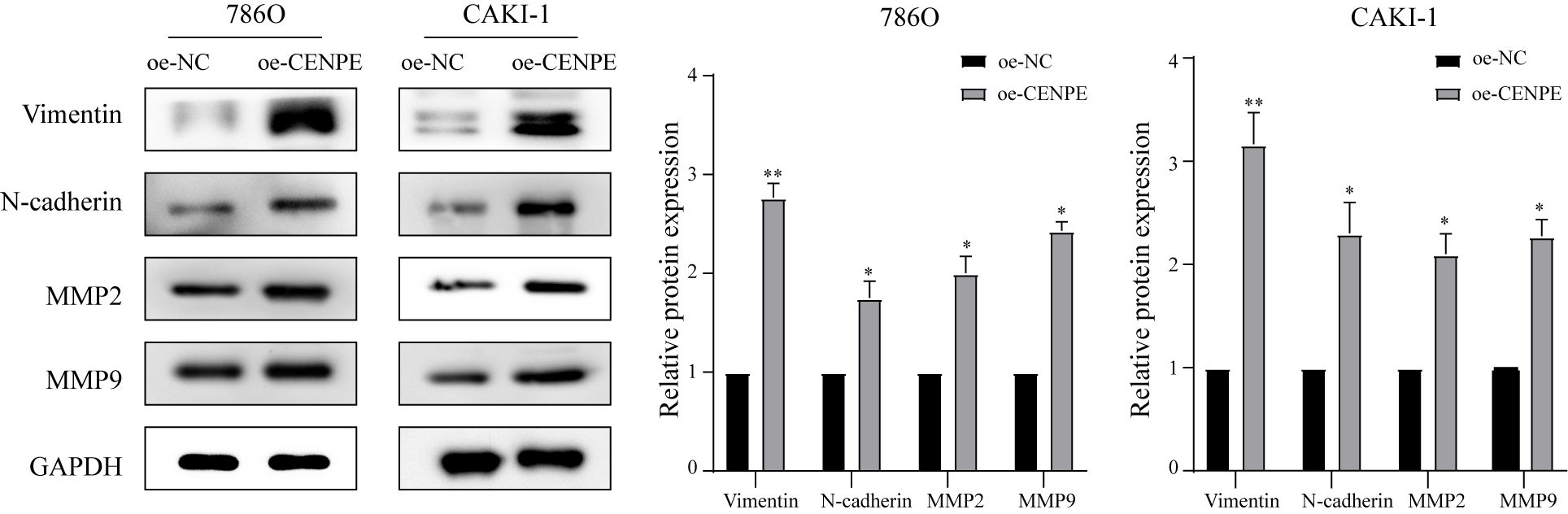

Supplement: Supplementary file 1 — Figure S1: Protein expressions of Vimentin, N‐cadherin, MMP2 and MMP9 in 786O and CAKI‐1 cells transfected with oe‐CENPE, analysed by Western blot. *p < 0.05, **p < 0.01. [file JCMM-29-e70773-s001.tif]

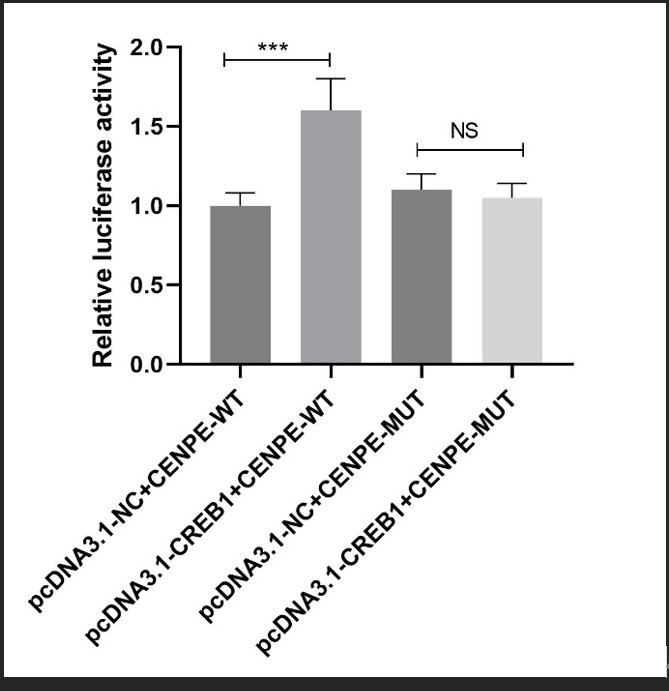

Supplement: Supplementary file 2 — Figure S2: Fluorescence activity in 786O cells with CENPE wildtype and mutant promotor with CREB1 pcDNA3. (***p < 0.001). [file JCMM-29-e70773-s002.jpg]
